# A physicochemical assessment of the thermal stability of dextrin–colistin conjugates

**DOI:** 10.1038/s41598-021-89946-2

**Published:** 2021-05-19

**Authors:** Emilie Chiron, Mathieu Varache, Joana Stokniene, David W. Thomas, Elaine L. Ferguson

**Affiliations:** 1 Advanced Therapies Group, Oral and Biomedical Sciences, School of Dentistry, College of Biomedical and Life Sciences, Cardiff University, Heath Park, Cardiff, CF14 4XY UK; 2 Present Address: CNRS, Ingénierie Des Matériaux Polymères, IMP UMR CNRS 5223, Université de Lyon, Université Claude Bernard Lyon 1, 15 bd A. Latarjet, 69622 Villeurbanne, France

**Keywords:** Drug delivery, Antibiotics, Pharmaceutics, Drug development, Translational research

## Abstract

Attachment of polysaccharide carriers is increasingly being used to achieve precision delivery and improved effectiveness of protein and peptide drugs. Although it is clear that their clinical effectiveness relies on the purity and integrity of the conjugate in storage, as well as following administration, instability of polysaccharide-based conjugates can reduce the protective efficacy of the polymer, which may adversely affect the bioactive’s potency. As a model, these studies used dextrin–colistin conjugates, with varying degrees of polymer modification (1, 2.5 and 7.5 mol% succinoylation) to assess the effect of storage temperature (− 20, 4, 21 and 37 °C) and duration (up to 12 months) on saccharide and colistin release and antimicrobial activity. Estimation of the proportion of saccharide release (by comparison of area under the curve from size exclusion chromatograms) was more pronounced at higher temperatures (up to 3 and 35% at − 20 °C and 37 °C, respectively after 12 months), however, repeated freeze–thaw did not produce any measurable release of saccharides, while addition of amylase (20, 100, 500 IU/L) caused rapid release of saccharides (> 70% total within 24 h). At all temperatures, conjugates containing the lowest degree of succinoylation released the highest proportion of free colistin, which increased with storage temperature, however no trend in saccharide release was observed. Despite the clear physical effects of prolonged storage, antimicrobial activity of all samples was only altered after storage at 37 °C for 12 months (> threefold decreased activity). These results demonstrate significant release of saccharides from dextrin–colistin conjugates during prolonged storage in buffered solution, especially at elevated temperature, which, in most cases, did not affect antimicrobial activity. These findings provide vital information about the structure–activity relationship of dextrin–colistin conjugates, prior to full-scale commercial development, which can subsequently be applied to other polysaccharide-protein and -peptide conjugates.

## Introduction

Since the 1970s, polysaccharides such as dextran, dextrin, hyaluronic acid, hydroxyethyl starch and polysialic acid have been investigated as carriers for therapeutic protein and peptide drugs in a wide range of clinical applications, including cancer, arthritis, viral infections, and, more recently, tissue repair and bacterial infection^1^. Polysaccharides possess many favorable features for the targeted delivery of protein and peptide drugs since they are biodegradable, hydrophilic and non-toxic. Given the reported potential of non-biodegradable polymers, such as polyethylene glycol and polyvinylpyrrolidone, to induce lysosomal storage disease and induce antibody formation^2–5^, the use of polysaccharides for the delivery of therapeutic proteins and peptides has become increasingly attractive. However, unlike well-defined conventional small molecule drugs, polysaccharide-protein and -peptide conjugates are complex mixtures that are not easily separated, identified or characterized. The clinical effectiveness of conjugated proteins and peptides relies on the purity and integrity of the conjugate in storage and following administration, as well as effective enzyme-triggered restoration of activity at the target site. Instability of polymer conjugates can reduce the protective efficacy of the polymer, which may adversely affect the protein or peptide’s potency.

Previous studies have shown enhanced drug stability when colistin (also known as polymyxin E) was conjugated to dextrin^6^ and alginate oligomer^7^. Dextrin, a linear polymer of D-glucose with over 95% α-1,4 links, was chosen as the carrier for the prototype polysaccharide-polymyxin conjugates because it is rapidly degraded in the body by α-amylase via random degradation of the polyglucose chain to form intermediate oligosaccharides and, ultimately, the disaccharides maltose and iso-maltose^8^. Thus, dextrin–colistin conjugates were designed to protect the antibiotic and mask its biological activity during passive targeting to sites of infection, while amylase degradation at the target site can reinstate colistin’s antibacterial activity. Modification of the dextrin backbone has been shown to delay payload release by amylase degradation of dextrin^6,9,10^. Preliminary stability studies have been performed in water and phosphate buffer solution (PBS) at 4 and 37 °C using size exclusion chromatography (SEC) to characterize the size and relative molecular weight of the conjugated colistin component. However, analysis of the polysaccharide’s degradation components has not yet been studied. Typically, polysaccharide-protein conjugates, synthesized using non-specific conjugation methods, contain complex mixtures of various lengths of oligosaccharides and unconjugated polysaccharide, as well as polydisperse conjugates with different conjugation efficiencies and polymer: peptide ratios; making them notoriously challenging to characterize. Quantification of released saccharides and colistin will help identify suitable storage conditions and support quality control of polysaccharide conjugates, to ensure compliance with manufacturing specifications and batch-to-batch consistency.

This study assessed the thermostability of dextrin–colistin conjugates containing dextrin with 1, 2.5 and 7.5 mol% succinoylation, in terms of saccharide and colistin release. For comparison, colistimethate sodium (CMS, also known as colistin methanesulfonate, Colomycin^®^) and amylase-degraded conjugates were also analyzed. Since variation in the purity and integrity of polymer-protein and -peptide conjugates may affect their potency, antimicrobial activity of antibiotic solutions after storage for 12 months at varying temperatures was also determined.

## Results

### Thermostability during storage

**Figure 1:**
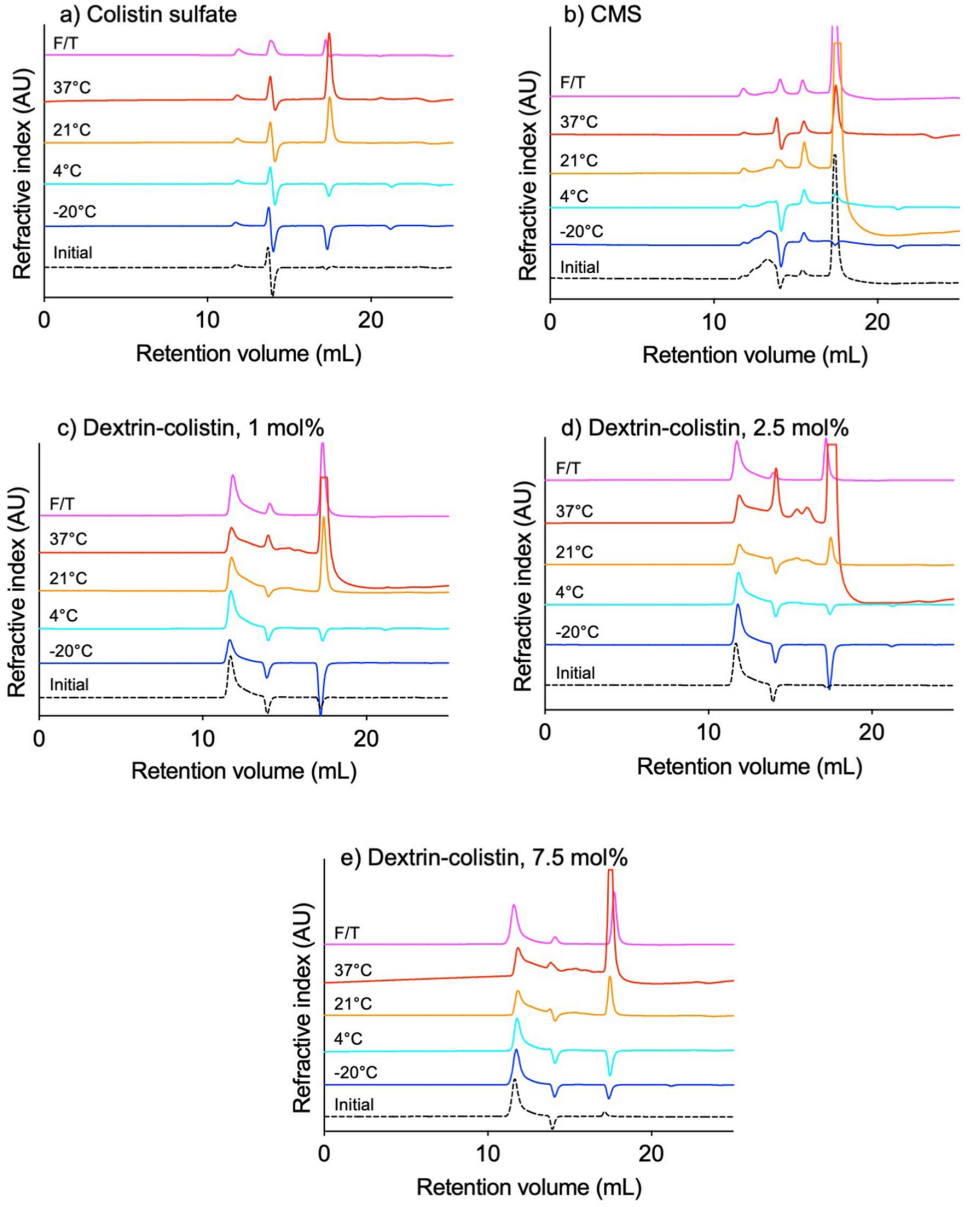
GPC chromatograms showing refractive index (RI) of (**a**) colistin sulfate, (**b**) colistimethate sodium (CMS) and dextrin–colistin conjugates with (**c**) 1 mol%, (**d**) 2.5 mol% and (**e**) 7.5 mol% succinoylation before (dotted line) and following storage at − 20 (dark blue line), 4 (light blue line), 21 (yellow line) or 37 (red line)°C for 12 months, or exposed to weekly freeze-thawing for 5 weeks (pink line).

**Figure 2:**
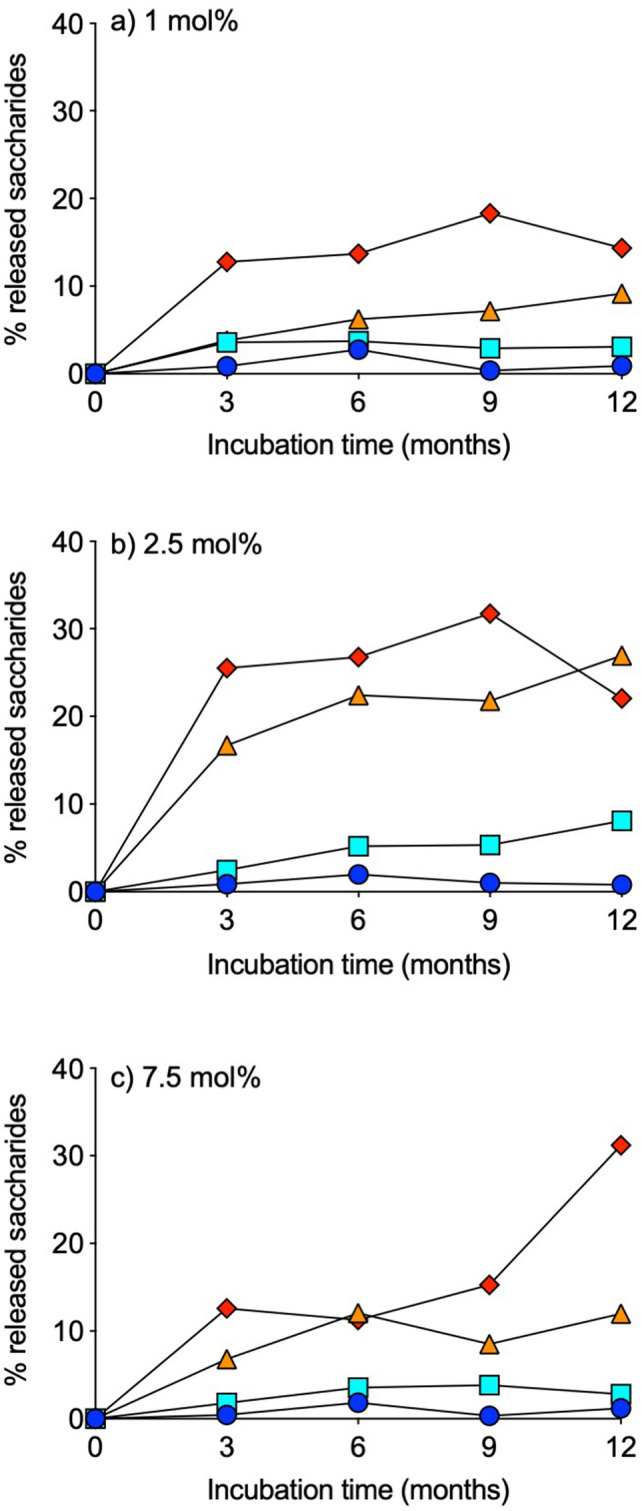
Release of free saccharides from dextrin–colistin conjugates with (**a**) 1 mol%, (**b**) 2.5 mol% and (**c**) 7.5 mol% succinoylation following storage at − 20 (dark blue circle), 4 (light blue square), 21 (orange triangle) or 37 (red diamond)°C for up to 12 months.

To assess the integrity and size of dextrin–colistin conjugates during storage, size exclusion chromatography with RI and UV absorbance (at 210 and 280 nm) detection was performed using FPLC and GPC systems. When stored samples were eluted through an Ultrahydrogel 120 column, colistin sulfate showed a small peak at ~ 12 mL and a larger peak at ~ 14 mL (Fig. 1, S1). Apart from an increased intensity of the peak corresponding to salts (~ 17.5 mL), colistin’s elution profile was largely unchanged after storage. Significant changes in the elution profile of CMS were observed after storage above − 20 °C. dextrin–colistin conjugate samples had a characteristically similar elution profile, with a large peak of high molecular weight material eluting in the void volume (~ 12 mL), followed by a series of smaller peaks that appeared or grew larger during storage (Fig. 1, S1). The appearance of additional, low molecular weight peaks was more pronounced at higher temperatures. When the area under the curve of peaks with molecular weight < 800, corresponding to oligo- and disaccharides, was used to estimate the proportion of released saccharides, time- and temperature-dependent release of sugars was observed (Fig. 2). Higher saccharide release was observed from conjugates containing 2.5 mol% dextrin at all storage temperatures, compared to 1 and 7.5 mol% (Figs. 1, 2).

**Figure 3:**
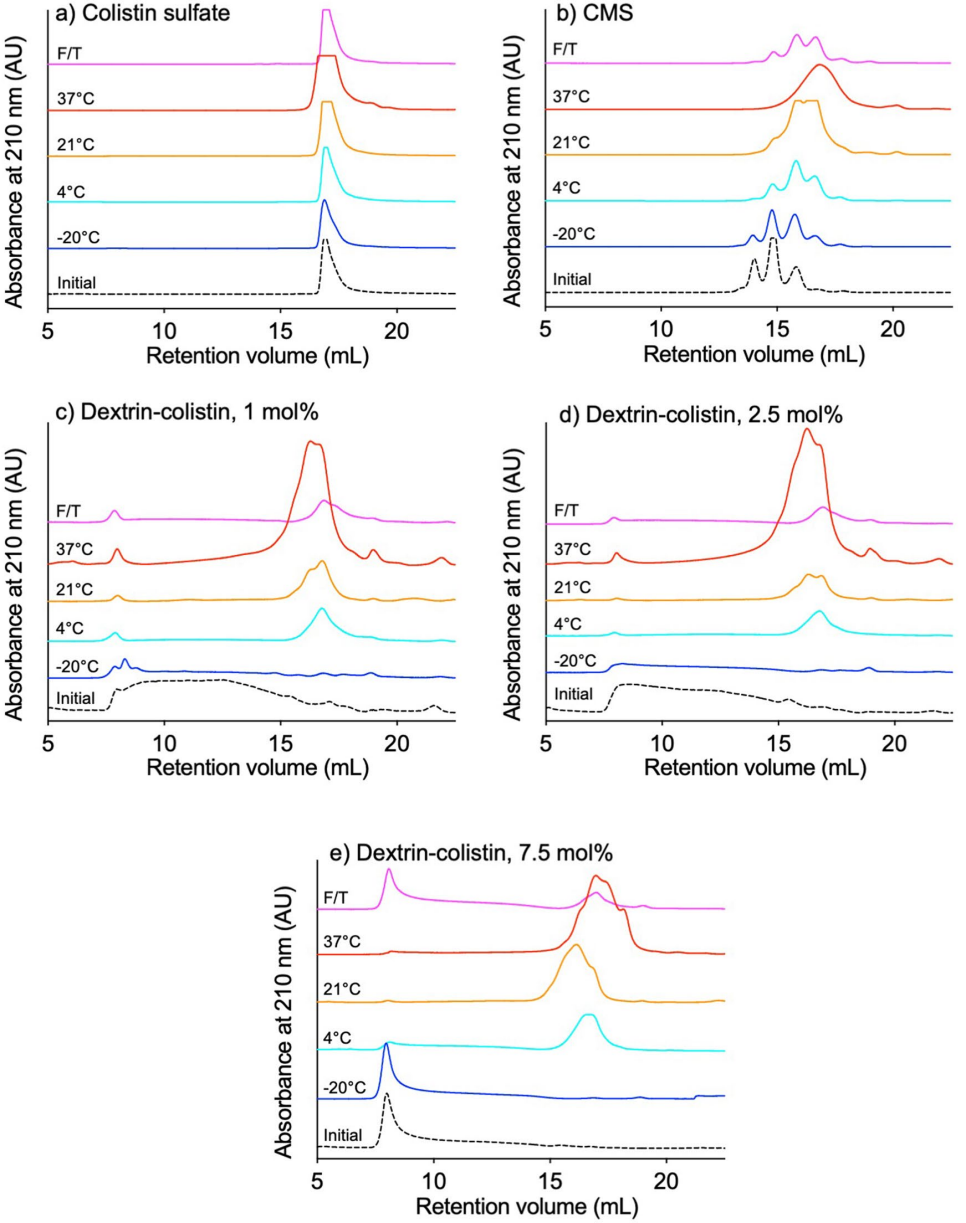
FPLC chromatograms showing absorbance at 210 nm of (**a**) colistin sulfate, (**b**) colistimethate sodium (CMS) and dextrin–colistin conjugates with (**c**) 1 mol%, (**d**) 2.5 mol% and (**e**) 7.5 mol% succinoylation before (dotted line) and following storage at − 20 (dark blue line), 4 (light blue line), 21 (yellow line) or 37 (red line)°C for 12 months or exposed to weekly freeze-thawing for 5 weeks (pink line).

**Figure 4:**
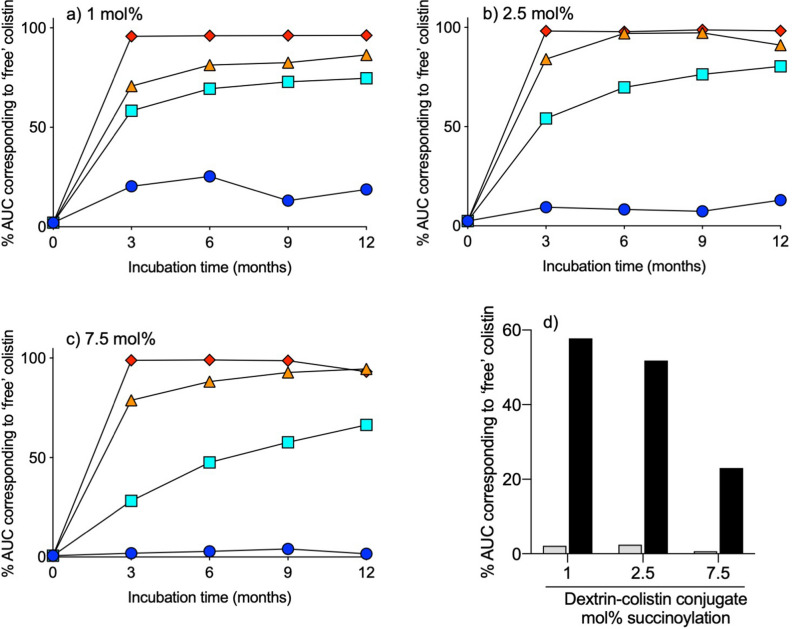
Release of free colistin from dextrin–colistin conjugates with (**a**) 1 mol%, (**b**) 2.5 mol% and (**c**) 7.5 mol% succinoylation following storage at -20 (dark blue circle), 4 (light blue square), 21 (orange triangle) or 37 (red diamond)°C for up to 12 months. Panel (**d**) shows release of free colistin from dextrin–colistin conjugates before and after exposure to weekly freeze-thawing for 5 weeks.

When stored samples were eluted through a Superdex 75 column, colistin sulfate gave a single peak at ~ 17 mL, while the elution profile of CMS had multiple peaks that changed in size during storage (Fig. 3). Dextrin–colistin conjugates had a broad peak that started in the void volume (~ 8 mL) and a second, small peak at the same elution volume as unmodified colistin (~ 17 mL). Conjugates containing dextrin with a higher degree of succinoylation had a more prominent peak in the void volume, while conjugates containing dextrin with a lower degree of succinoylation had a broader peak corresponding to the conjugate. Conjugate elution pattern was maintained when samples were stored at − 20 °C, while appearance or growth of a peak at ~ 17 mL was evident after storage at 4, 21 and 37 °C. When the area under the curve for the peak at ~ 17 mL, corresponding to free colistin, was used to estimate the proportion of released colistin, time- and temperature-dependent release of colistin was observed (Fig. 4). Under each storage condition, conjugates containing the lowest degree of succinoylation had the highest proportion of free colistin.

When dextrin–colistin conjugates were subjected to repeated freeze–thaw, no measurable release of saccharides was observed (Fig. 1). Nevertheless, a peak corresponding to free colistin appeared after repeated freeze–thaw and was largest for conjugates containing dextrin with the lowest degree of succinoylation (Fig. 4d).

Antimicrobial activity of the test compounds following repeated freeze–thaw or storage in solution for up to 12 months was assessed by minimum inhibitory concentration (MIC) assay. As observed previously, the antimicrobial activity of dextrin–colistin conjugates was lower than colistin sulfate and CMS, and decreased further with increasing degree of succinoylation (Table 1). Antimicrobial activity of colistin sulfate, CMS and the dextrin–colistin conjugates was unaffected by repeated freeze–thaw or storage in solution for up to 12 months at − 20, 4 or 21 °C. However, when antibiotic solutions were stored at 37 °C for 12 months, a significant decrease in antimicrobial activity was observed (> threefold).

**Table 1:** Antimicrobial activity of colistin sulfate, CMS and dextrin–colistin conjugates following storage at -20, 4, 21 or 37 °C for 12 months or exposed to weekly freeze-thawing for 5 weeks, measured by MIC assay. Data is expressed as mode (*n* = 3). MIC value represents equivalent colistin concentration of conjugates.

Bacterial strain	Treatment	MIC (mg/L)					
		Initial	Storage temperature (°C)				Repeat freeze–thaw
			*-20*	*4*	*21*	*37*	
*V5 E. coli* AIM-1	Colistin sulfate	0.004	0.002	0.002	0.016	0.25	0.004
	CMS	0.125	0.031	0.063	0.063	8	0.031
	Dextrin–colistin 1 mol%	1	1	1	4	> 128	0.5
	Dextrin–colistin 2.5 mol%	1	2	1	4	> 128	0.5
	Dextrin–colistin 7.5 mol%	8	16	4	16	> 128	4
*V6 K. pneumoniae* IR25	Colistin sulfate	0.125	0.063	0.125	0.5	> 128	0.25
	CMS	2	1	1	1	128	0.5
	Dextrin–colistin 1 mol%	64	128	128	128	> 128	64
	Dextrin–colistin 2.5 mol%	> 128	> 128	> 128	> 128	> 128	64
	Dextrin–colistin 7.5 mol%	> 128	> 128	> 128	> 128	> 128	> 128
*V19 A. baumannii* 7789	Colistin sulfate	0.063	0.063	0.031	0.125	32	0.063
	CMS	0.5	0.25	0.125	0.25	64	0.125
	Dextrin–colistin 1 mol%	64	128	64	> 128	> 128	16
	Dextrin–colistin 2.5 mol%	128	128	64	> 128	> 128	32
	Dextrin-colistin 7.5 mol%	> 128	> 128	> 128	> 128	> 128	128

### Degradation of dextrin-colistin conjugates by amylase

**Figure 5:**
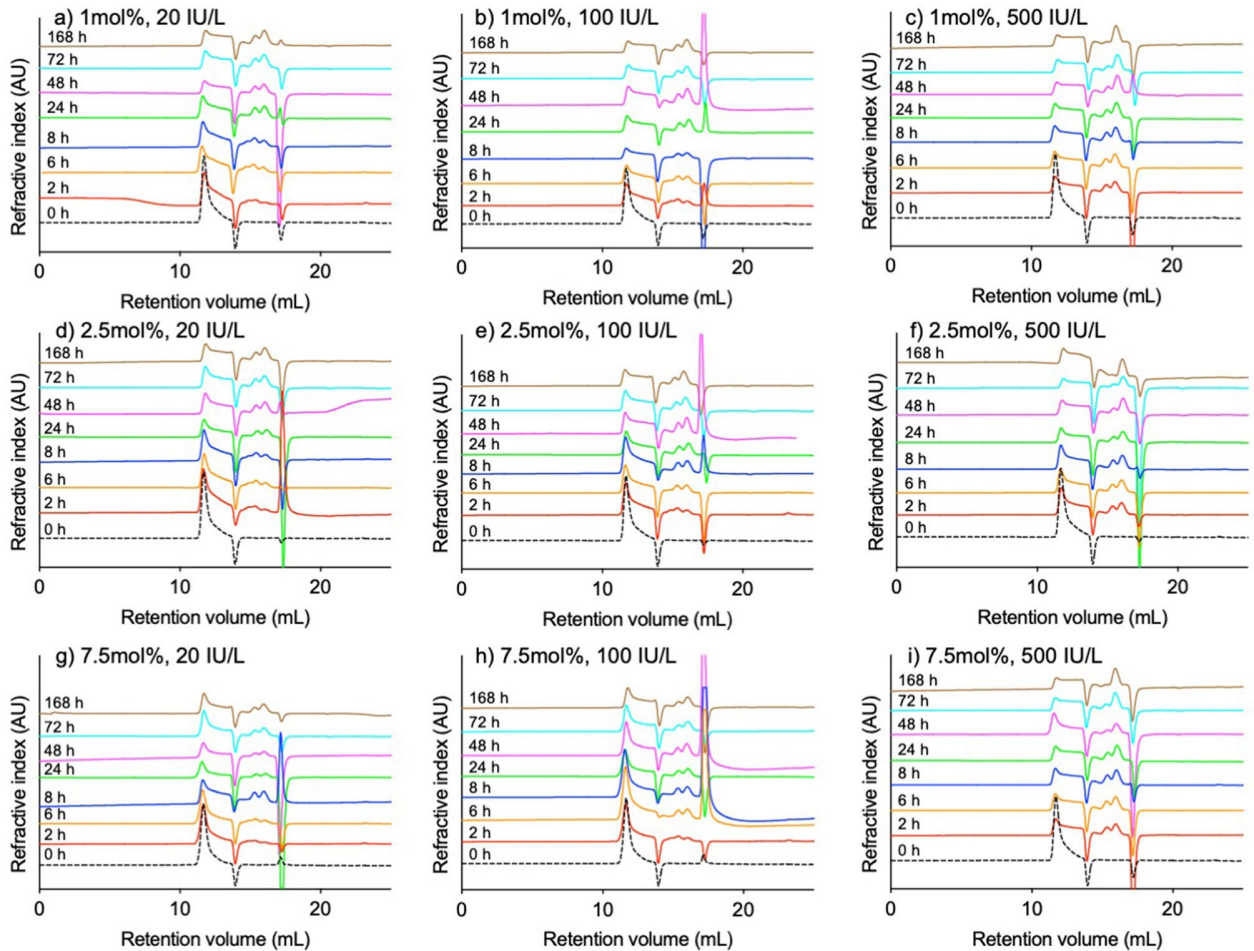
GPC chromatograms showing refractive index (RI) of dextrin-colistin conjugates with (**a**-**c**) 1 mol%, (**d**-**f**) 2.5 mol% and (**g**-**i**) 7.5 mol% succinoylation following incubation with amylase (0, 20, 100 and 500 IU/L) at 37 °C for 0 (dotted line), 2 (red line), 6 (yellow line), 8 (dark blue line), 24 (green line), 48 (pink line), 72 (light blue line) and 168 (brown line) h.

**Figure 6:**
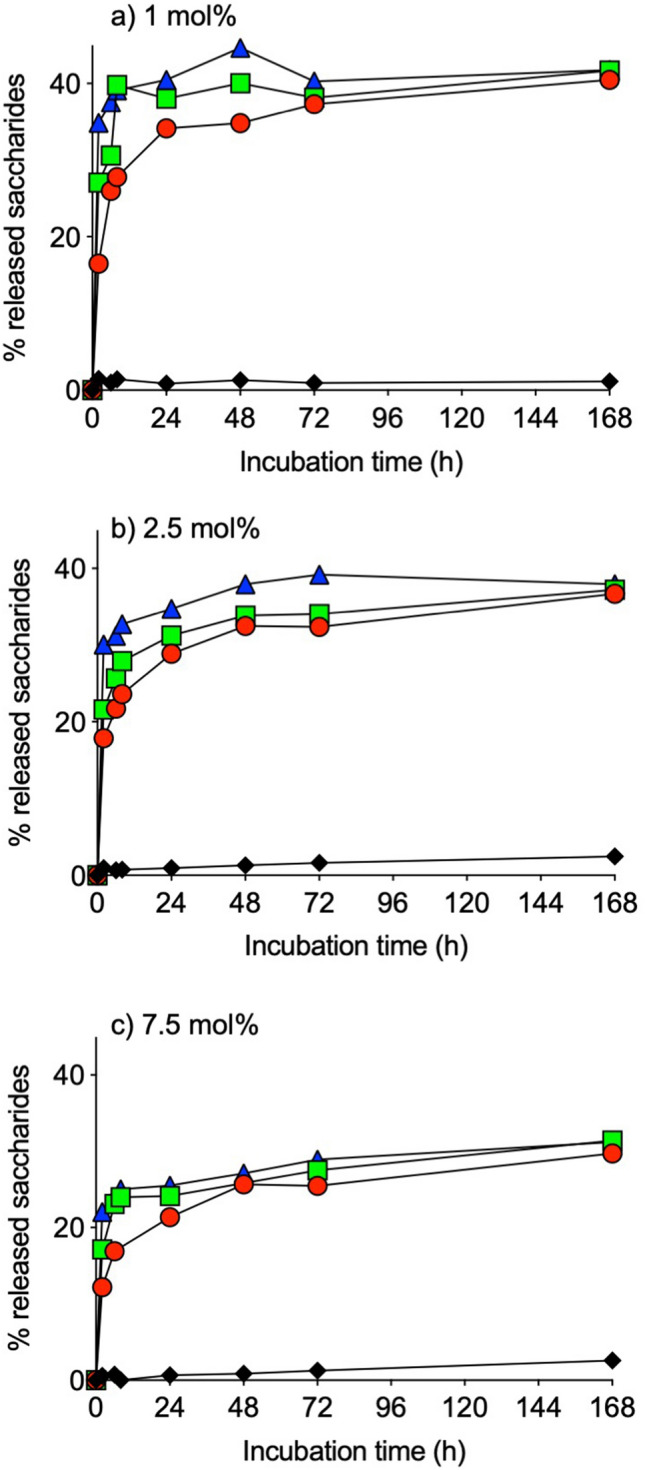
Release of free saccharides from dextrin-colistin conjugates with (**a**) 1 mol%, (**b**) 2.5 mol% and (**c**) 7.5 mol% succinoylation following incubation with amylase (0 (black diamond), 20 (red circle), 100 (green square) and 500 (dark blue triangle) IU/L) at 37 °C for up to 168 h.

Release of saccharides during amylase-degradation of the dextrin-colistin conjugates was assessed using GPC (Fig. 5). Less than 2.6% of total AUC corresponded to free saccharides when conjugates were incubated at 37 °C for up to 168 h in the absence of amylase, however, addition of amylase caused rapid release of saccharides, which was greatest for conjugates containing dextrin with the lowest degree of succinoylation (Fig. 6). Increasing amylase concentration from 20 to 500 IU/L led to modestly greater total saccharide release, but increased the proportion of smaller oligosaccharides and disaccharides (Fig. 5).

## Discussion

Studies to investigate the stability of colistin and CMS in aqueous media have, to date, mostly been limited to just a few days or weeks^11–13^. Here, we have investigated the thermostability of colistin sulfate, CMS (a complex mixture of methane sulfonated colistin derivatives) and dextrin-colistin conjugates during long-term storage in buffered solution.

Dextrin-colistin conjugates were developed, in part, to overcome the spontaneous hydrolysis of CMS to partial sulfomethyl derivatives and colistin in aqueous solution, which may result in accidental administration of elevated levels of colistin. For this reason, it is recommended that CMS should be reconstituted in sterile water no more than 24 h prior to administration^14^. Here, hydrolysis of CMS during storage in PBS buffer, even at 4 °C and after repeated freeze–thaw, was evident from the altered FPLC elution profiles. Although previous studies have used much shorter storage durations they agree that CMS hydrolyses faster at higher temperatures and in salt solutions^11–13^.

In this study, dextrin-colistin conjugates were dissolved and stored in PBS at pH 7.4, since this is a common diluent for in vitro experiments and it is representative of the pH of tissues it would reside in after intravenous (IV) administration. This is also close to the optimum pH for α-amylase optimal enzyme activity (6.7–7.0)^15^. Degradation of polysaccharides in alkaline solution is well known, generally occurring at the reducing end of the polymer and proceeding stepwise along the chain^16^. Studies show that alkaline degradation of glycans containing 14 linkages, such as dextrin, produces isosaccharinates, following a rearrangement to a ketose form^16^. Although temperature is known to affect the speed of polysaccharide degradation, it does not affect the nature or proportion of the degradation products and there is typically minimal degradation at low temperatures (< 25 °C)^16–18^. Even though pH 7.4 is only slightly alkaline, significant degradation of dextrin was observed during prolonged incubation of dextrin-colistin conjugates in PBS buffer at elevated temperatures. GPC chromatograms of conjugate samples stored for up to 12 months show the appearance of several peaks with molecular weights corresponding to maltotetraose (667), maltotriose (504) and maltose (360). As storage temperature and duration increased, the peaks corresponding to maltose and smaller oligosaccharides became more prominent, suggestive of further degradation of larger oligosaccharides and polymers. Unexpectedly, conjugates containing 2.5 mol% succinoylation released more saccharides than those containing 1 or 7.5 mol% succinoylated dextrin. The reason for this is unclear and does not appear to be related to concentration, drug loading, molecular weight or size. We hypothesize that the spacing between succinoylated sugars may affect the rate and extent of dextrin degradation, whereby the steric effects of 2.5 mol% succinoylation is optimal for enzyme activity. Alternatively, the 2.5 mol% succinoylation may create an optimum pH in the microenvironment at the surface of the polymer which maximizes amylase’s catalytic rate by influencing enzymatic substrate accumulation and equilibrium constants of the reaction. Further studies to confirm and investigate this observation are ongoing. The presence of an alkali-resistant linkage in the polysaccharide can interrupt alkaline degradation before complete degradation is achieved^18^, however it does not appear that succinoylation has a noticeable effect on alkaline degradation under the conditions tested here.

After IV administration, dextrin-colistin conjugates would be exposed to α-amylase, an endoamylase that cleaves dextrin within the polyglucose chain, in the bloodstream. Previous studies have shown that the antimicrobial activity of dextrin-colistin conjugates was greatest when colistin was attached to shorter chains of dextrin with minimal modification by succinoylation^19^. Here, we have shown rapid concentration-dependent release of low molecular weight oligosaccharides and maltose in the presence of α-amylase, which was greatest for conjugates with the lowest degree of succinoylation. After an initial burst of saccharide release, the total proportion of released sugars from 1, 2.5 and 7.5 mol% conjugates reached a plateau at ~ 41, ~ 37 and ~ 30%, respectively. This supports the premise that residual chains of dextrin attached to colistin in conjugates with higher degrees of succinoylation obstruct its antimicrobial activity^19^.

Detection of colistin can be achieved using several methods, including chromatography with fluorescence after derivatization^20–23^ or mass spectrometry^19,24–27^ detection, microbiological assays^28,29^, enzyme-linked (ELISA)^6,30^ and derivative spectrophotometry^31^. Here, analysis of FPLC chromatograms was achieved by monitoring absorbance at 210 nm. Storage of conjugate solutions above 4 °C caused an increase in the amount of free colistin detected, which increased with storage temperature and was lowest for conjugates containing dextrin with higher degrees of succinoylation. Analysis of the area under the curve of peaks corresponding to dextrin-colistin conjugate and free colistin suggests that almost complete release of colistin was reached within 3 month when conjugates were stored at 37 °C and within 12 months when they were stored at 21 °C. However, even for colistin sulfate and CMS solutions, the total AUC of all peaks increased with storage time and temperature (Fig. 3), meaning that these estimates are unreliable. It is unclear why absorbance at 210 nm by the stored solutions increased over time and at increased temperature. Analysis of the same samples by GPC showed a large peak corresponding to high molecular weight conjugate by RI (Fig. 1, S1), as well as absorbance at 210 nm (*data not shown*) detection, indicating the presence of high molecular weight species containing both dextrin and colistin, even after 12 months storage. It is unlikely that solute evaporation caused the observed increase in colistin’s UV absorbance at higher storage temperatures, because no noticeable volume reduction was observed from tightly sealed tubes. Instead, it is reasonable to assume that prolonged storage of samples at 21 and 37 °C caused changes to colistin’s secondary structure that altered its UV absorption. Alternatively, variation in counterion concentration can lead to increased UV absorbance. Previous studies have shown that UV absorbance of the peptide bond (200–230 nm) in a 33.5 μM α-synuclein solution decreased with increasing counterion concentration^32^.

Reassuringly, despite apparent degradation of dextrin-colistin conjugates during storage above 4 °C, antimicrobial activity was only reduced when conjugates were stored for 12 months at 37 °C. This trend was also observed for colistin sulfate and CMS. Although relatively less degradation of the conjugates occurred after repeated freeze–thaw of stock solutions (compared to during incubation with amylase or after prolonged storage above 4 °C), this was not associated with diminished antimicrobial activity. In this study, it was not possible to obtain MIC values above 128 mg/L as stored samples were prepared at 3 mg/mL conjugate, which limited the maximum colistin concentration that could be tested. However, in agreement with previous studies^6^, antimicrobial activity of dextrin-colistin conjugates decreased with increasing degree of dextrin succinoylation. It is not clear, from this study or from previous research, whether degradation of dextrin-colistin conjugates occurs during the incubation period of MIC assay experiments. Previous studies showed that pre-incubation of conjugates with amylase at equivalent concentrations as that found in human serum (100 IU/L) did not alter their antimicrobial potency^5^. Although the bacterial strains tested here do not secrete amylase (measured by Phadebas assay, *data not shown*), it is likely that other metabolic enzymes in the culture medium or secreted by the bacteria, as well as incubation at 37** °C** for 16–20 h, would, at least partially, degrade dextrin. When Varache et al.^19^ tested the antimicrobial activity of differentially degraded fractions of dextrin-colistin conjugates, they found that antimicrobial activity was greatest for fully ‘unmasked’ conjugates, so further degradation of the conjugates during the MIC assay, or in vivo, at sites of inflammation/ infection, would be expected to enhance its antimicrobial activity.

In clinical practice, it is unlikely that antibiotic solutions would be stored for prolonged periods at elevated temperatures, as used in this study. Lyophilized samples of dextrin-colistin conjugates are stable for at least 12 months, with no effect on structural stability or antimicrobial potency (*data not shown).* All dextrin-colistin conjugates were stable when stored in solution at − 20 °C for up to 12 months, however repeat freeze–thaw resulted in detectable levels of released colistin. These findings suggest that aliquots of stock solutions of conjugates can be safely stored at or below − 20 °C for up to 12 months, however, repeat freeze–thaw should be avoided.

This study has developed and validated a method to test the thermostability of dextrin-colistin conjugates, which can subsequently be applied to other polysaccharide-protein and -peptide conjugates. This methodology will provide crucial understanding of the rate and extent of saccharide release during storage and in physiological solutions containing amylase. This study has verified the purity and integrity of dextrin-colistin conjugates currently in pre-clinical development, prior to full-scale commercial development.

## Materials and methods

### Materials

Type I dextrin from corn (M_w_ = 7500), colistin sulfate, α-amylase from human saliva, *N*-hydroxysulfosuccinimide (sulfo-NHS), 3-(4,5-dimethylthiazol-2-yl)-2,5-diphenyl tetrazolium bromide (MTT), bicinchoninic acid (BCA) solution, dimethyl sulfoxide (DMSO), glucose and maltose oligomers (DP 2, 3, 6 and 7) were purchased from Sigma-Aldrich (Poole, UK). 1-Ethyl-3-(3-(dimethylamino)propyl carbodiimide hydrochloride) (EDC) was acquired from Pierce (Rockford, USA). Disodium hydrogen phosphate, potassium dihydrogen phosphate, potassium chloride, 4-dimethylaminopyridine (DMAP), and sodium chloride were from Fisher Scientific (Loughborough, UK). Unless otherwise stated, all chemicals were of analytical grade and used as received. All solvents were of general reagent grade (unless stated) and were from Fisher Scientific (Loughborough, UK). Colistimethate sodium (CMS, Colomycin®) was from Teva UK Limited (Eastbourne, UK).

#### Bacterial culture

The clinical isolates (V5 *Escherichia coli* AIM-1, V6 *Klebsiella pneumoniae* IR25 and V19 *Acinetobacter baumannii* 7789) and susceptibility testing method have been previously described by Khan et al.^33^ Bacterial colonies were grown on blood agar supplemented with 5% v/v defibrinated horse blood. Overnight cultures were prepared in tryptone soy broth (TSB) and Mueller–Hinton broth (MHB) was used for MIC determination (LabM; Bury, UK).

#### Synthesis of dextrin-colistin conjugates

Dextrin-colistin conjugates, having 1, 2.5 and 7.5 mol% succinoylation of dextrin (Mw = 7500; degree of polymerization (DP) = 50), were synthesized using EDC and sulfo-NHS, purified by fast protein liquid chromatography (FPLC) using a HiLoad 16/600 Superdex 75 column and characterized according to previously optimized methods^11^. Molecular weight was determined using a Viscotek TDA302 triple detector system, equipped with integrated refractive index (RI), viscometer and light scattering (LALS and RALS) detection and a Viscotek 2501 UV detector (210 nm). The column set consisted of an Ultrahydrogel guard column and two Ultrahydrogel 250 columns (7.8 mm × 300 mm) from Waters (Elstree, UK) in series, running at 30 °C with PBS buffer eluent and a flow rate of 0.7 mL/min. Samples (300 μL) were injected into a 100 μL loop. A polyethylene oxide standard (Mw = 23,964, Mn = 23,502, IV = 0.404) from Malvern Panalytical (Malvern, UK) was employed for the calibration set up. The characteristics of dextrin-colistin conjugates used in these studies are summarized in Table 2.

**Table 2:** Characteristics of dextrin-colistin conjugates used in these studies.

Conjugate	Mw^a^ (M_w_/M_n_)	R_H_^a^ (nm)	Protein content (% w/w)	Molar ratio (x dextrin: 1 colistin)	Free protein (%)
Dextrin-colistin 1 mol%	29,600 (1.6)	2.6	5.8	2.9	2.1
	21,700 (1.7)	2.5	7.4	2.2	7.0
Dextrin-colistin 2.5 mol%	30,300 (1.5)	2.7	6.4	2.6	2.4
	23,000 (1.8)	2.6	9.3	1.7	4.5
Dextrin-colistin 7.5 mol%	59,800 (1.8)	3.2	14.0	1.1	0.7
	74,900 (3.5)	3.5	18.1	0.8	1.5

^a^ measured using Viscotek TDA 302 detector with 2 × Ultrahydrogel 250 columns.

### Stability sample preparation

#### Stability during storage

Three primary batches of colistin sulfate, CMS and dextrin-colistin conjugates were dissolved in PBS at 3 mg/mL and stored at − 20, 4, 21 and 37** °C** for 0, 1, 3, 6, 9 and 12 months. In addition, to test the effect of repeated freeze–thaw on stability, a separate set of solutions were stored at − 20** °C** and defrosted for 1 h at 20–22** °C** weekly for 5 cycles. Samples were analyzed using FPLC (free drug) and GPC (saccharide release) and antimicrobial activity, UV/vis absorbance and protein content (bicinchoninic acid (BCA) assay) of the samples stored for 12 months was assessed.

#### Fast protein liquid chromatography

FPLC was performed using an ÄKTA Purifier from GE Healthcare (Amersham, UK) column with a UV detector and data analysis using Unicorn 5.31 software from GE Healthcare (Amersham, UK). Samples (200 μL) were injected into a 100 μL loop at 0.5 mL/min, connected to a prepacked Superdex 75 10/300 GL column with PBS buffer eluent. The proportion of free colistin was calculated by integrating the peaks corresponding to conjugate and free drug.

#### Gel permeation chromatography

GPC was performed using an Ultrahydrogel guard column and two Ultrahydrogel 120 columns (7.8 mm × 300 mm) from Waters (Elstree, UK) in series, connected to a Jasco PU-1580 HPLC pump with PBS buffer eluent and a flow rate of 1 mL/min. Samples (300 μL) were injected into a 100 μL loop and the eluate was monitored using a Jasco MD-2010 multi-wavelength UV detector (210 and 280 nm) and a Gilson 153 differential refractometer (Middleton, WI, USA) connected to a Polymer Laboratories PL DataStream (Church Stretton, UK). Dual detection enabled distinction of dextrin and saccharide components that are not bound to colistin. Cirrus GPC software (version 3.2, 2006) from Polymer Laboratories (Church Stretton, UK) was used for data analysis. Glucose and maltose oligomers (DP 2, 3, 6 and 7) were employed for calibration. Area under the curve of peaks corresponding to maltose, maltotriose and maltotetraose were used to calculate the proportion of free saccharides, compared to total AUC of all peaks with molecular weight > 300.

#### Antimicrobial activity

The minimum inhibitory concentration (MIC) of colistin sulphate, CMS and dextrin-colistin conjugates was determined using the broth microdilution method in MH broth in accordance with standard guidelines^34^. Test organisms were suspended in MH broth (100 µL, 5 × 10^5^ colony forming units (CFU)/mL) and incubated in 96-well microtitre plates in serial two-fold dilutions of the test compounds. The MIC was defined as the lowest concentration of test compound that produced no visible growth after 16–20 h. Results were expressed as mode (n = 3).

#### Degradation of dextrin-colistin conjugates by amylase.

Dextrin-colistin conjugates were dissolved in PBS (3 mg/mL colistin base in PBS, pH 7.4) containing amylase (0, 20, 100 and 500 IU/L) and incubated at 37 °C for 0, 2, 6, 12, 24, 48, 72, 168 h. Samples were analyzed using FPLC and GPC, as described previously.

## Supplementary Information

Supplementary Information 1.
