# Supplementary figures and images for: A physicochemical assessment of the thermal stability of dextrin–colistin conjugates
Source: Sci Rep. 2021 May 19;11:10600. doi: 10.1038/s41598-021-89946-2 (PMC8134461; doi:10.1038/s41598-021-89946-2)

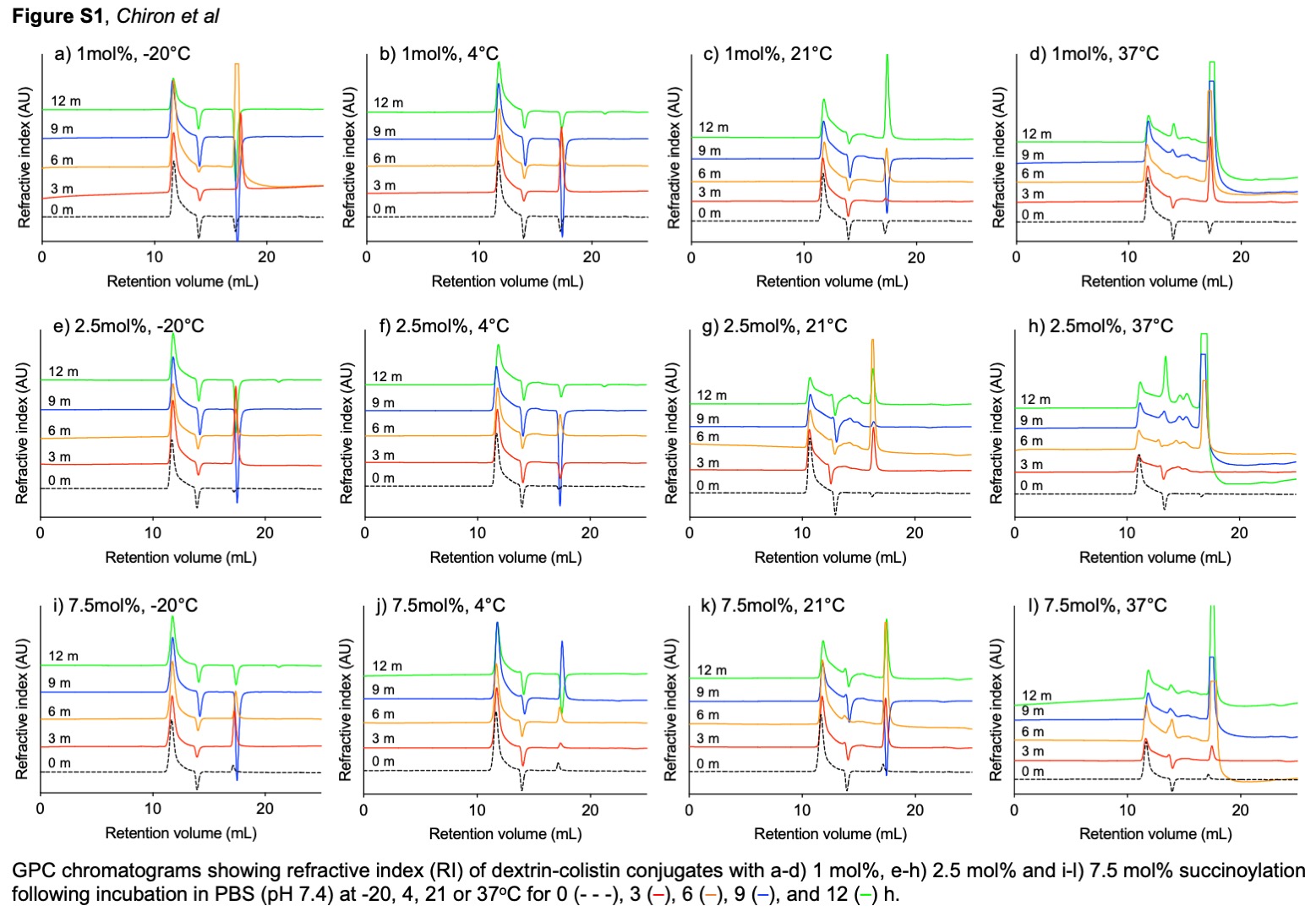

Supplement: Supplementary file 1 — Supplementary Information 1. [file 41598_2021_89946_MOESM1_ESM.jpeg]
